# Supplementary material for: Identification of Differential N-Glycan Compositions in the Serum and Tissue of Colon Cancer Patients by Mass Spectrometry
Source: Biology (Basel). 2021 Apr 20;10(4):343. doi: 10.3390/biology10040343 (PMC8074232; doi:10.3390/biology10040343)
Supplement: Supplementary file 1 [file biology-10-00343-s001.zip › Supp4_Figures_S1-S17.pdf]

Identification of differential N-glycans in the serum and tissue of colon cancer patients by mass spectrometry

Marcelo de Melo Andrade Coura[1,2*], Eder Alves Barbosa[2,3], Guilherme Dotto Brand[3], Carlos Bloch Jr[2], and Joao Batista de Sousa[1]

[1]Division of Colorectal Surgery, University Hospital of Brasilia, School of Medicine, University of Brasilia, Brasilia-DF, Brazil
[2]Laboratory of Mass Spectrometry, EMBRAPA Genetic Resources and Biotechnology, Brasilia-DF, Brazil
[3]Laboratory for the Synthesis and Analysis of Biomolecules, Institute of Chemistry, University of Brasilia, Brasilia-DF, Brazil

Supplemental material 4

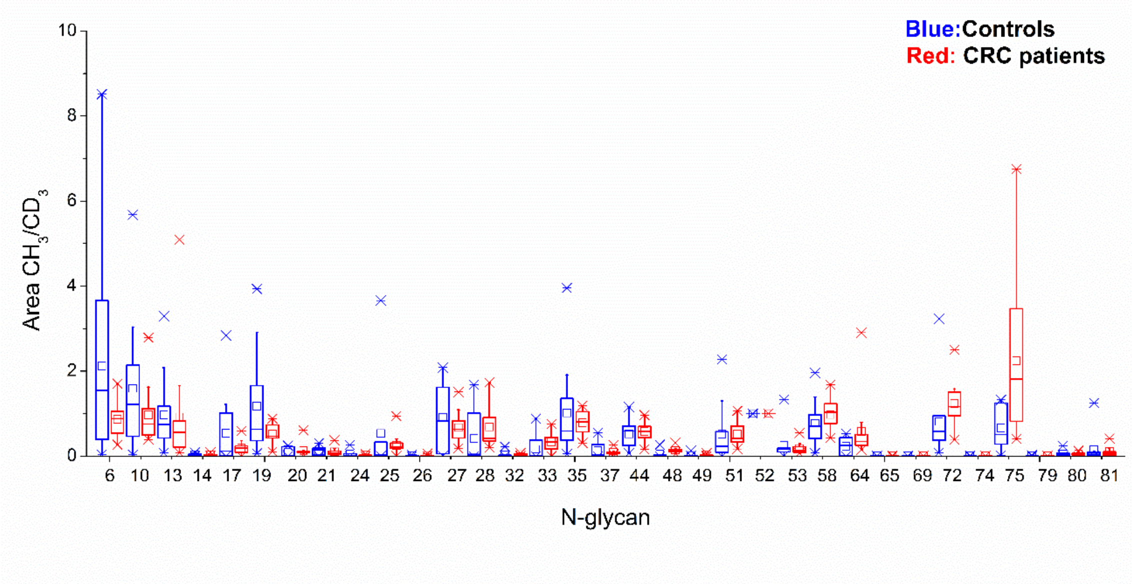

Figure S1. Box plots showing relative abundance N-glycans isolated from serum of normal and colon cancer patients analysed by MALDI MS. The lower and upper whiskers represent the 5th and 95th percentile, respectively, while the median is represented as a straight line, the mean by an open box (□) and data outliers are represented by the X symbol.

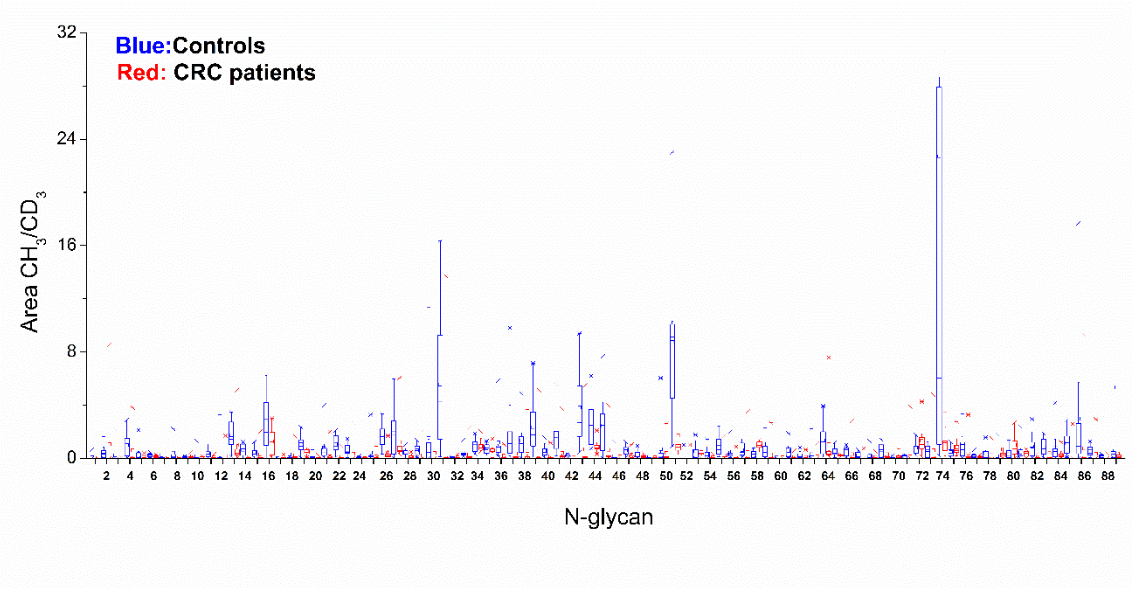

Figure S2. Box plots showing relative abundance N-glycans isolated from serum of normal and colorectal CRC patients analysed by LC/MS. The lower and upper whiskers represent the 5th and 95th percentile, respectively, while the median is represented as a straight line, the mean by an open box (□) and data outliers are represented by the X symbol.

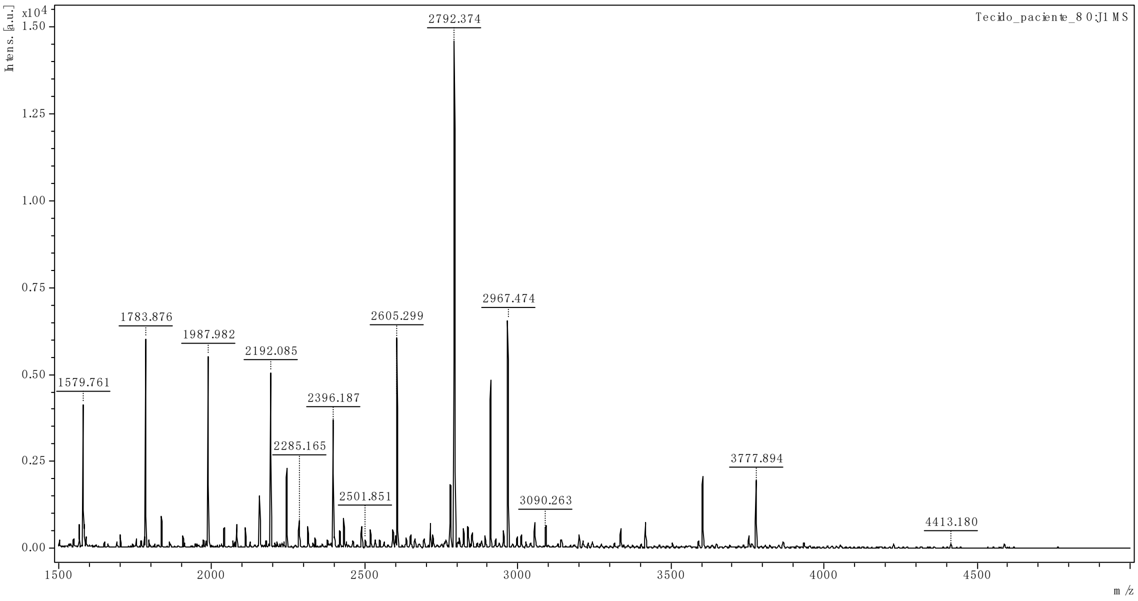

Figure S3. MALDI/MS acquisition of N-glycans extracted from normal and colon cancer tissues of patient 1: male, 37 years-old, transverse tumor, stage II, CEA 1.48, KRAS mutation +. Ion signals [M+Na^+].

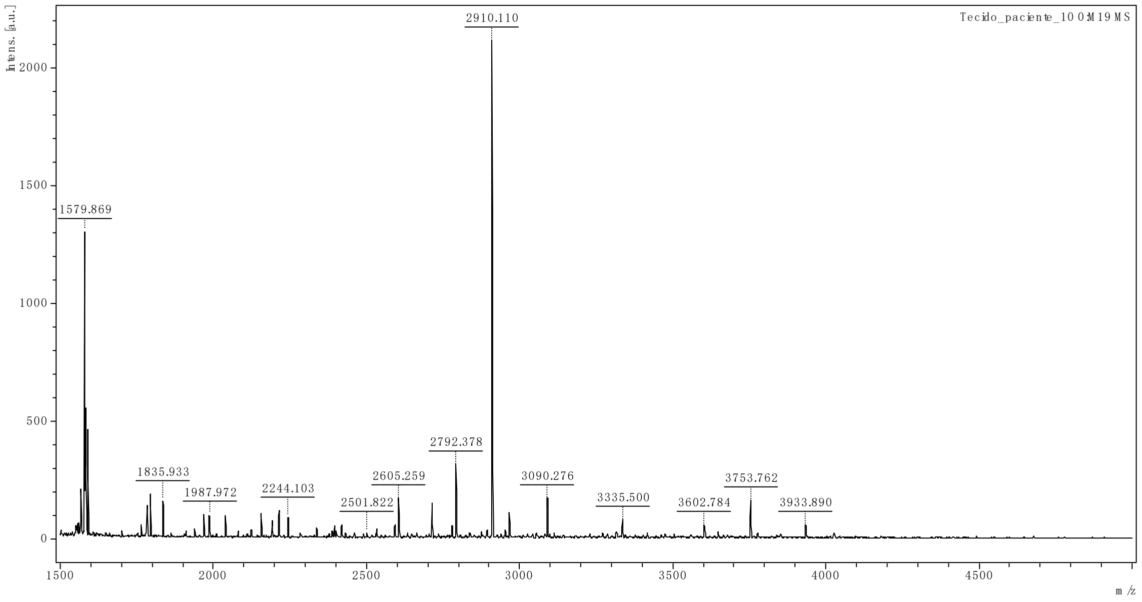

Figure S4. MALDI/MS acquisition of N-glycans extracted from normal and colon cancer tissues of patient 2: female, 36 years-old, sigmoid tumor, stage II, CEA 1.65, no mutation. Ion signals [M+Na$^+$].

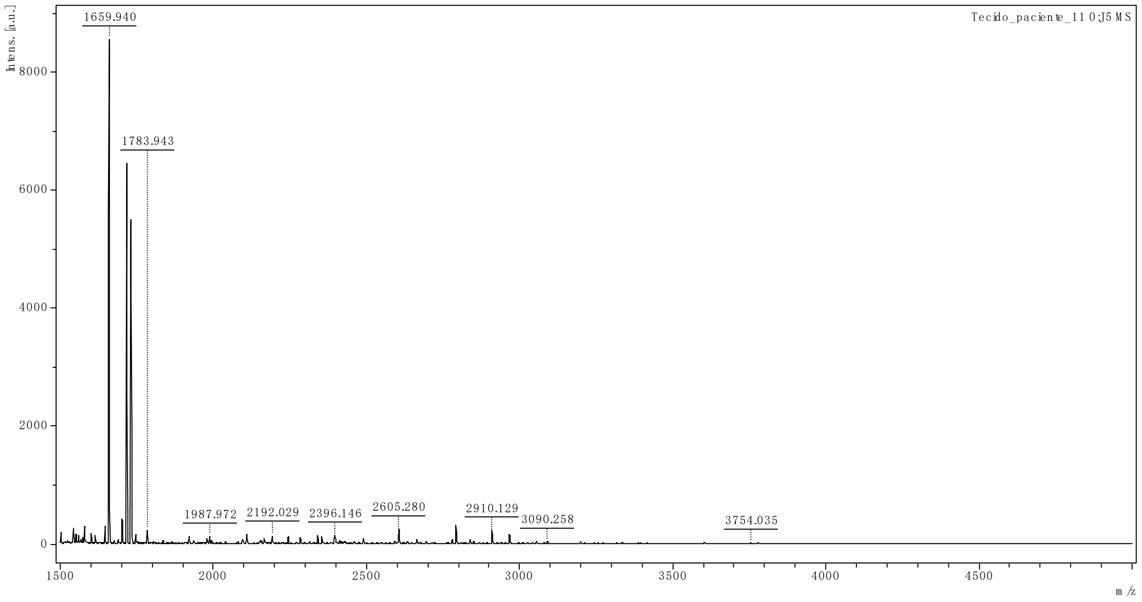

Figure S5. MALDI/MS acquisition of N-glycans extracted from normal and colon cancer tissues of patient 3: male, 65 years-old, cecum tumor, stage II, CEA 1.6, KRAS mutation +. Ion signals [M+Na$^+$].

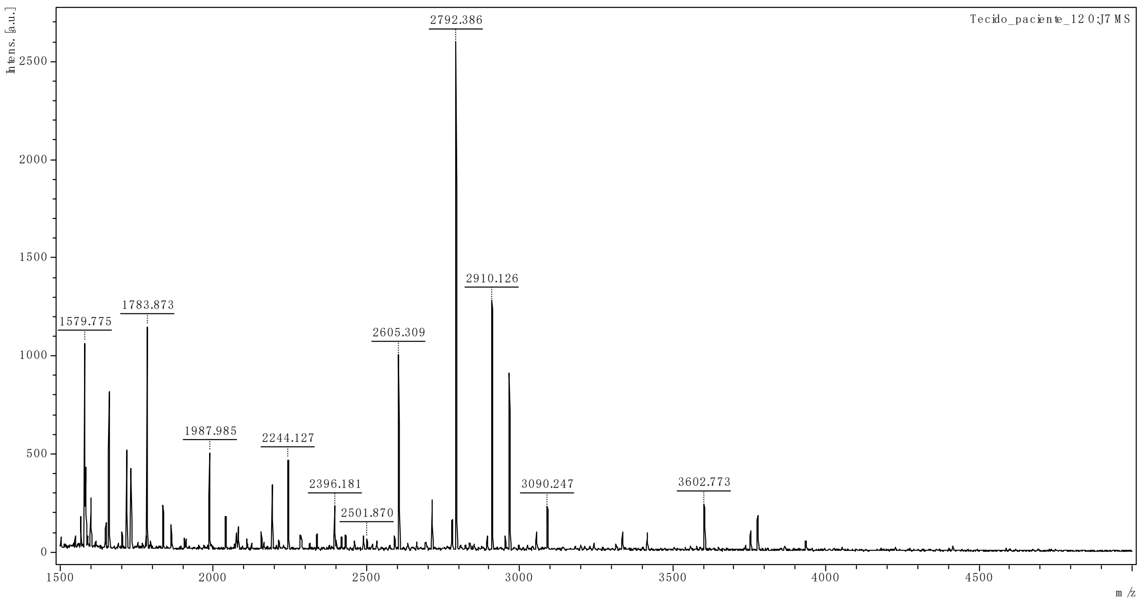

Figure S6. MALDI/MS acquisition of N-glycans extracted from normal and colon cancer tissues of patient 4: male, 55 years-old, descending tumor, stage III, CEA 5.3, no mutation. Ion signals [$M+Na^+$].

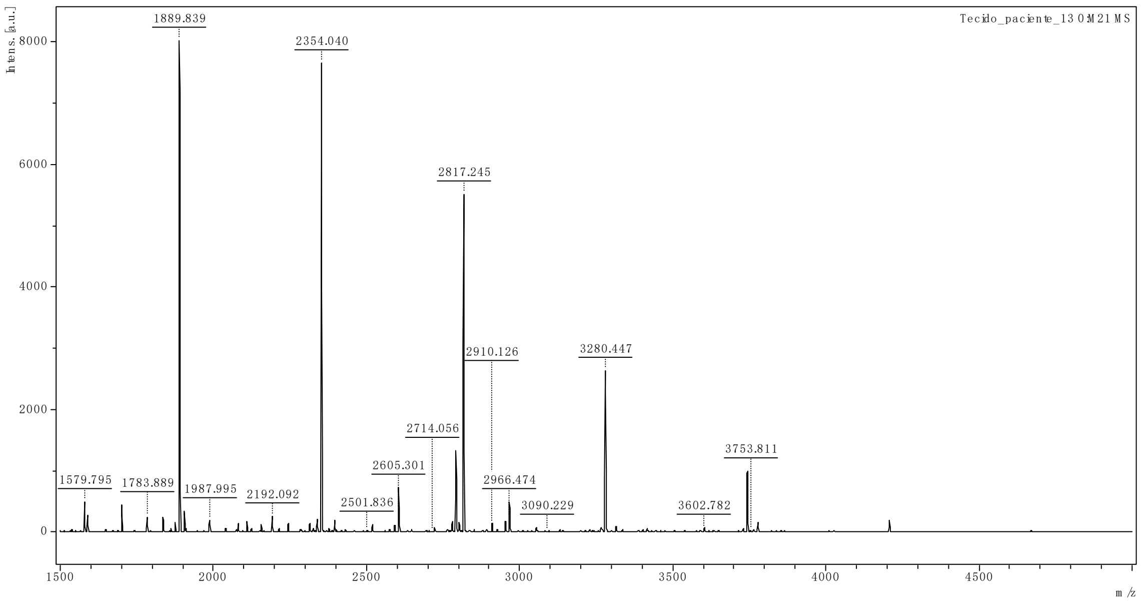

Figure S7. MALDI/MS acquisition of N-glycans extracted from normal and colon cancer tissues of patient 5: male, 60 years-old, sigmoid tumor, stage II, CEA 7.7, no mutation. Ion signals [$M+Na^+$].

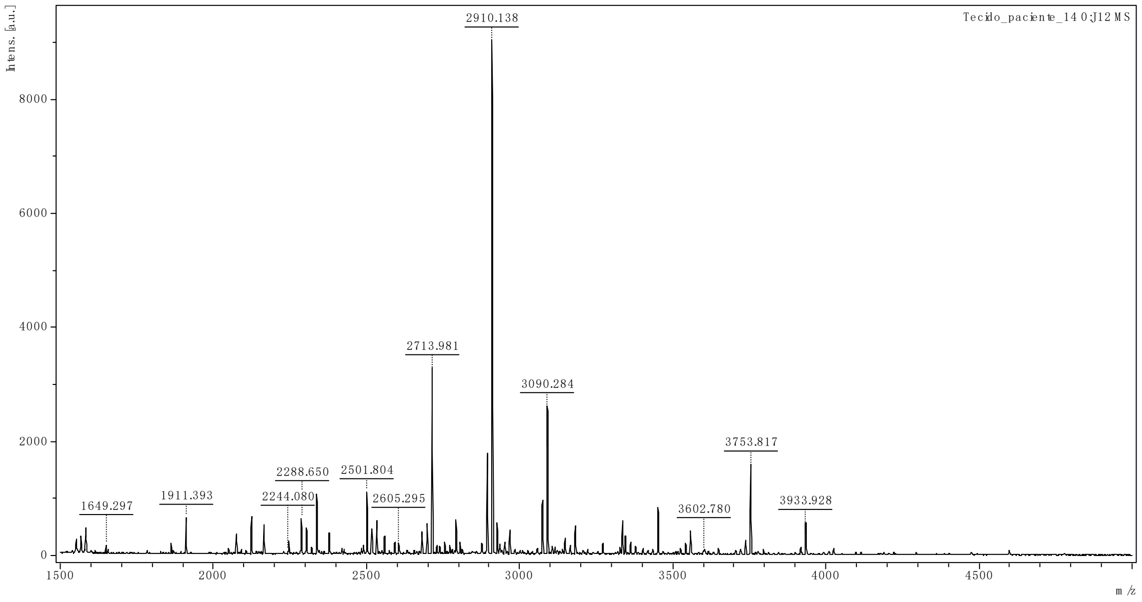

Figure S8. MALDI/MS acquisition of N-glycans extracted from normal and colon cancer tissues of patient 6: female, 56 years-old, sigmoid tumor, stage III, CEA 2.1, no mutation. Ion signals [M+Na$^+$].

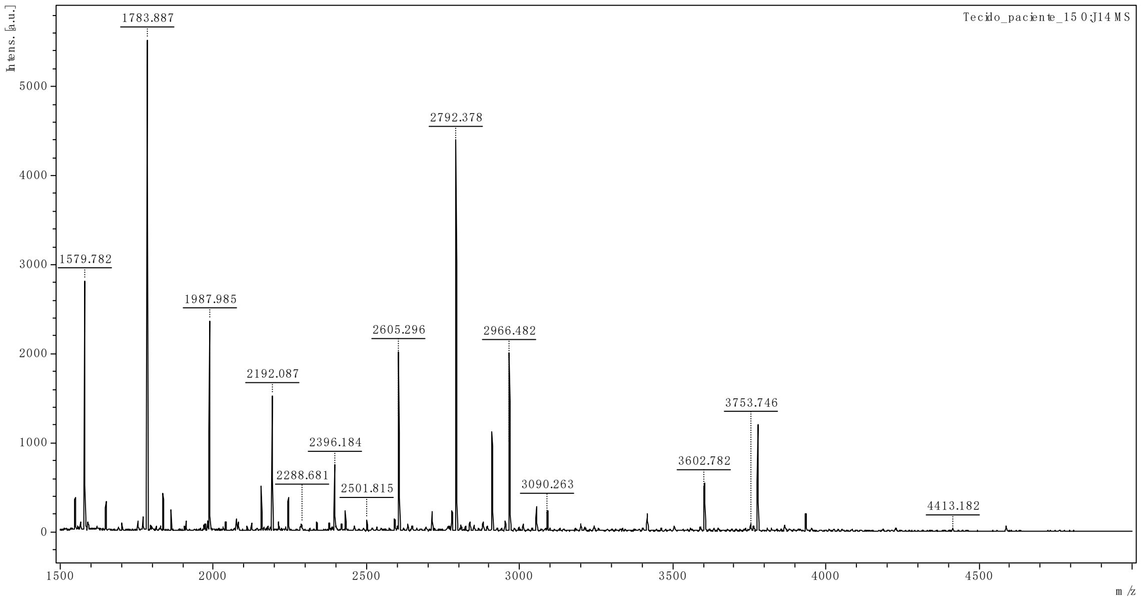

Figure S9. MALDI/MS acquisition of N-glycans extracted from normal and colon cancer tissues of patient 7: male, 67 years-old, ascending tumor, stage III, CEA 2.3, BRAF mutation +. Ion signals [M+Na$^+$].

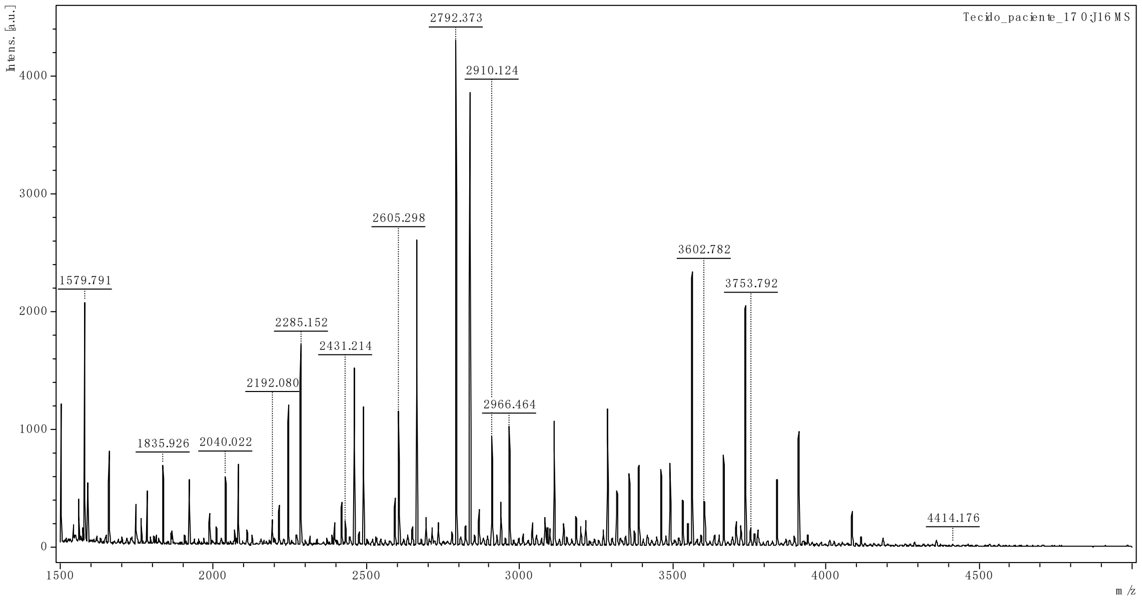

Figure S10. MALDI/MS acquisition of N-glycans extracted from normal and colon cancer tissues of patient 8: female, 64 years-old, cecum tumor, stage III, CEA 11.25, no mutation. Ion signals $[M+Na^{+}]$.

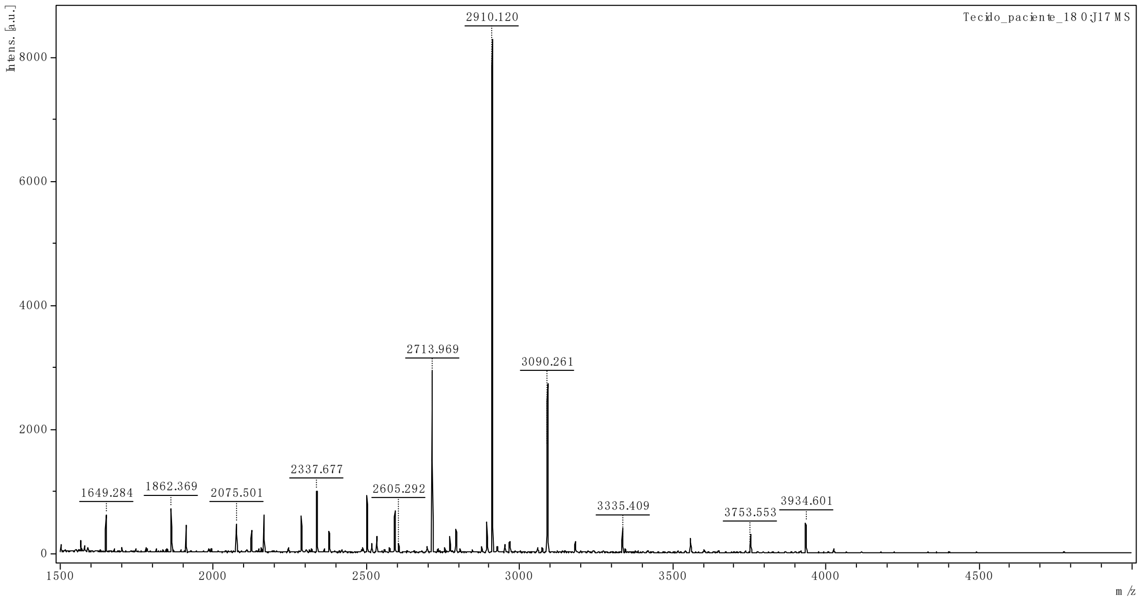

Figure S11. MALDI/MS acquisition of N-glycans extracted from normal and colon cancer tissues of patient 9: female, 63 years-old, cecum tumor, stage II, CEA 6.51, no mutation. Ion signals $[M+Na^{+}]$.

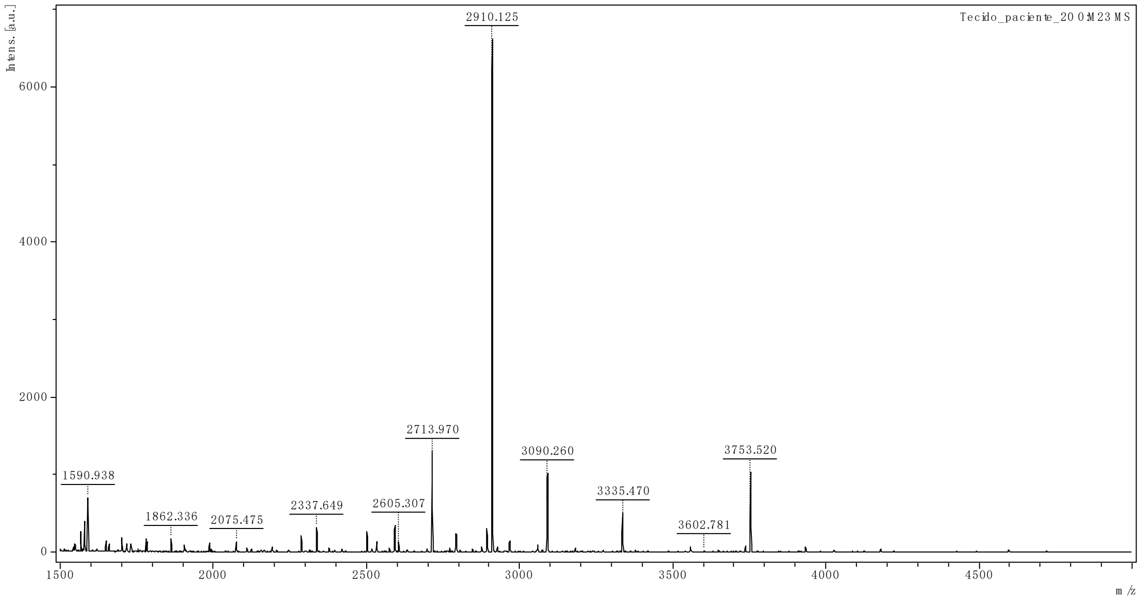

Figure S12. MALDI/MS acquisition of N-glycans extracted from normal and colon cancer tissues of patient 10: female, 54 years-old, sigmoid tumor, stage III, CEA 3.3, BRAF mutation +. Ion signals $[M+Na^{+}]$.

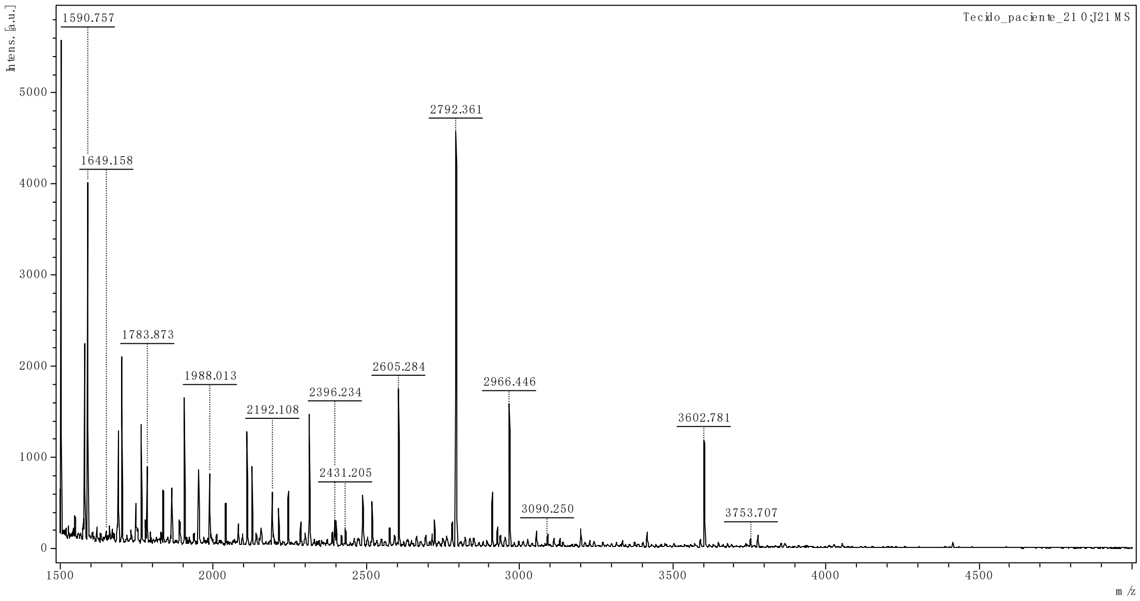

Figure S13. MALDI/MS acquisition of N-glycans extracted from normal and colon cancer tissues of patient 11: female, 74 years-old, sigmoid tumor, stage III, CEA 6.75, no mutation. Ion signals $[M+Na^{+}]$.

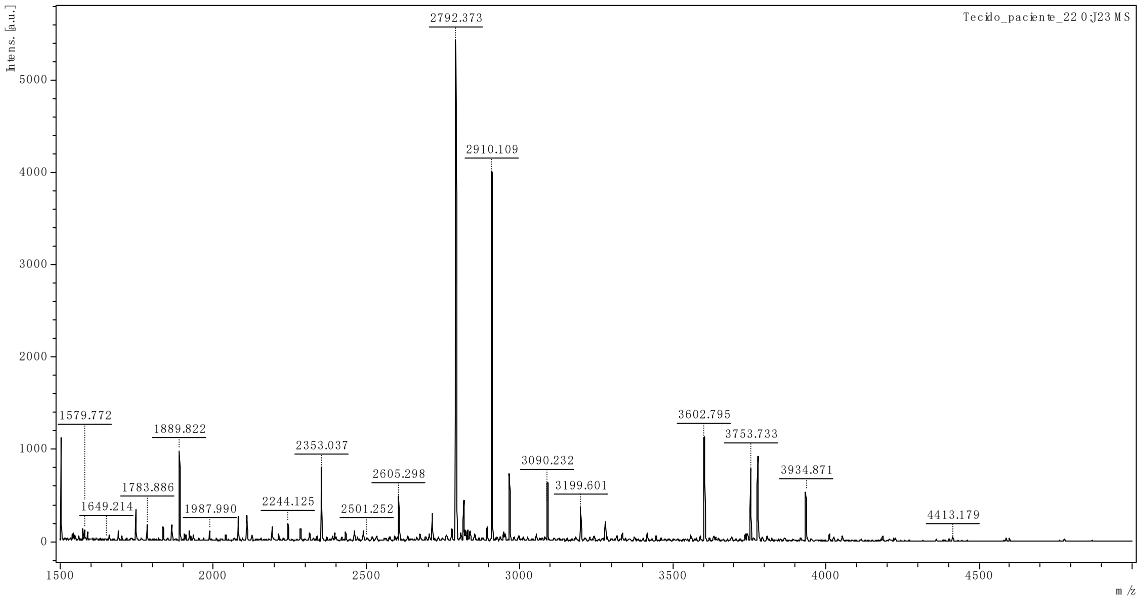

Figure S14. MALDI/MS acquisition of N-glycans extracted from normal and colon cancer tissues of patient 12: male, 64 years-old, cecum tumor, stage III, CEA 5.2, KRAS mutation +. Ion signals [M+Na$^+$].

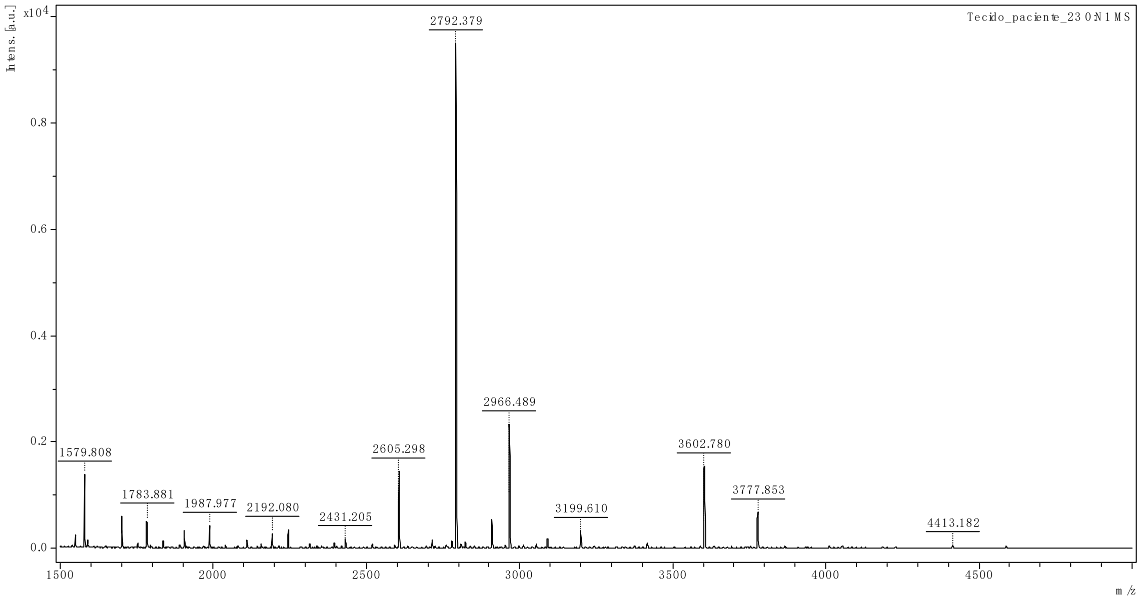

Figure S15. MALDI/MS acquisition of N-glycans extracted from normal and colon cancer tissues of patient 11: female, 75 years-old, sigmoid tumor, stage II, CEA 1.35, KRAS mutation +. Ion signals [M+Na$^+$].

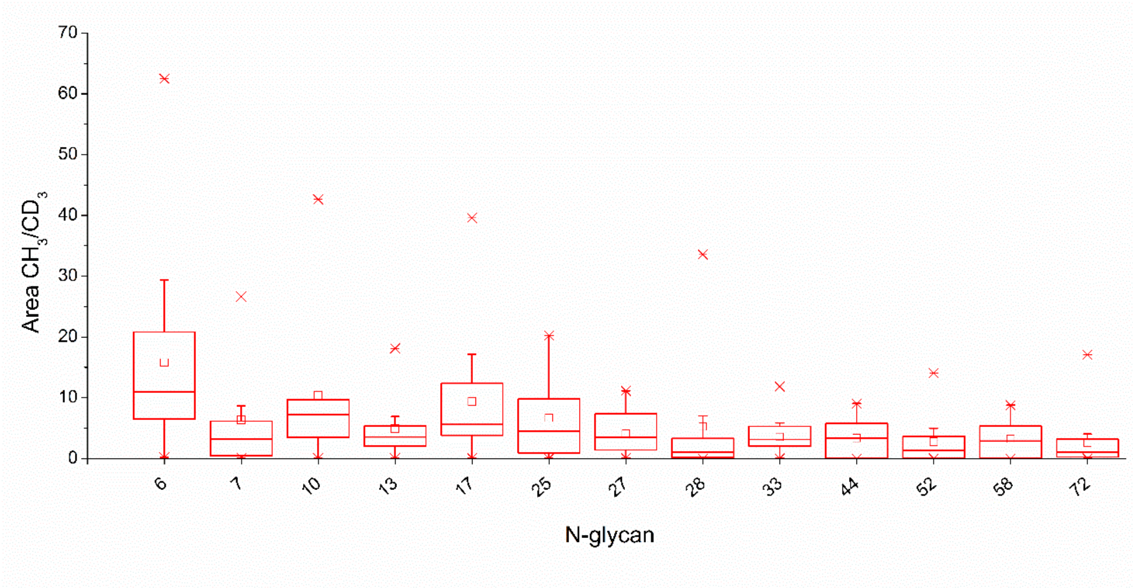

Figure S16. Box plots showing relative abundance N-glycans isolated from normal and colon cancer tissues analysed by MALDI MS. The lower and upper whiskers represent the 5th and 95th percentile, respectively, while the median is represented as a straight line, the mean by an open box (□) and data outliers are represented by the X symbol.

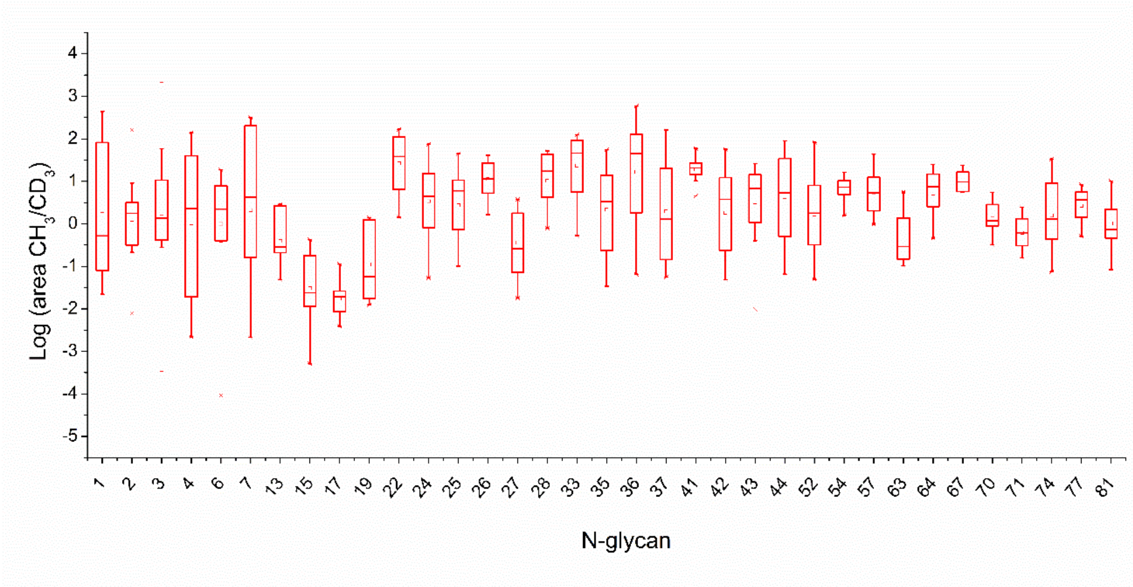

Figure S17. Box plots showing relative abundance N-glycans isolated from normal and colon cancer tissues analysed by LC-MS. The lower and upper whiskers represent the 5th and 95th percentile, respectively, while the median is represented as a straight line, the mean by an open box (□) and data outliers are represented by the X symbol.
