# Supplementary material for: Identification of Differential N-Glycan Compositions in the Serum and Tissue of Colon Cancer Patients by Mass Spectrometry
Source: Biology (Basel). 2021 Apr 20;10(4):343. doi: 10.3390/biology10040343 (PMC8074232; doi:10.3390/biology10040343)
Supplement: Supplementary file 1 [file biology-10-00343-s001.zip › Suppl_material_5_Tables S4_S5.pdf]

Identification of differential N-glycans in the serum and tissue of colon cancer patients by mass spectrometry

Marcelo de Melo Andrade Coura[1,2*], Eder Alves Barbosa[2,3], Guilherme Dotto Brand[,3], Carlos Bloch Jr[2], and Joao Batista de Sousa[1]

[1]Division of Colorectal Surgery, University Hospital of Brasilia, School of Medicine, University of Brasilia, Brasilia-DF, Brazil
[2]Laboratory of Mass Spectrometry, EMBRAPA Genetic Resources and Biotechnology, Brasilia-DF, Brazil
[3]Laboratory for the Synthesis and Analysis of Biomolecules, Institute of Chemistry, University of Brasilia, Brasilia-DF, Brazil

Supplemental material 5

SERUM

Table S3. N-glycosylation modifications in serum of CRC patients

Method	Aiming	Finding	Composition	Reference
Lectin blot	Total serum N-glycans, immunodepleted plasma,CRC patients vs controls	Increase of sialylation and fucosylation	↑ 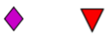	Qiu et al, 2008
Aleuria aurantia lectin blot	β-haptoglobin N-glycosylation, CRC patients, Chron's disease and controls	Higher AAL affinity in haptoglobin from CRC patients	↑ 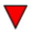	Park et al, 2010
Lectin blot	Total serum N-glycans, CRC patients vs controls	Decrease of core fucosylation	↓ 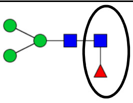	Zhao et al, 2011
Liquid chromatography (UPLC)	IgG N-glycosylation, CRC patients vs controls	Decrease of galactosylation and sialylation, increase of core fucosylation	↓ 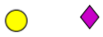 ↑ 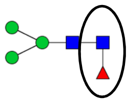	Vučković et al, 2016
Liquid chromatography (UPLC)	IgG N-glycosylation, Prognostic in CRC patients	Higher mortality with decrease of galactosylation and sialylation Increase of bisecting forms	↓ 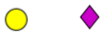 ↑ 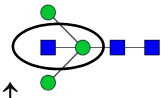	Theodoratou et al, 2016

MALDI-TOF/MS, electrophoresis	Total serum N-glycans, CRC patients vs controls	Increase of multi-antennae core- and outer-arm fucosylated	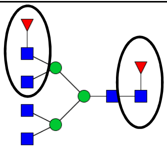<br>↑	Snyder et al, 2016
Liquid chromatography<br><br>(UPLC)	Total serum N-glycans, CRC patients vs controls	Decreased of core-fucosylated di-antennary asialo and monosialo,<br><br>Increase of multi-antennae sialylated	↓<br>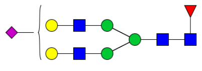<br>↑<br>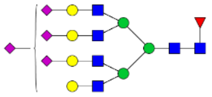	Doherty et al, 2018
MALDI-TOF/MS	Total serum N-glycans, prognostic in CRC patients	Decrease of core-fucosylated di-antennary<br><br>Increase of multi-antennae sialylated, sialyl Lewis	↓<br>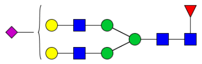<br>↑<br>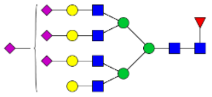<br>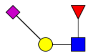<br>↑	de Vroome et al, 2018
MALDI-TOF/MS, LC/MS	Total serum N-glycans, CRC patiens vs controls	Increase of mannose -rich, bianntenary core fucosylated di-sialo and multi-antennae sialylated forms<br><br>decrease of galactosylated forms	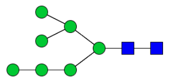<br>↑<br>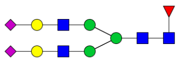<br>↑<br>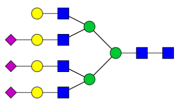<br>↑<br>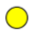<br>↓	present study, 2021

TISSUE

Table S4. N-glycosylation modifications in cell lines and tissues of CRC

Method	Aiming	Finding	Compositions	Reference
CRC tissues/Lectin	N-glycosylation profile in MUC1/CEACAM 5 glycoproteins	Increase of sialylated, high-mannose and branched N-glycans in CEACAM 5	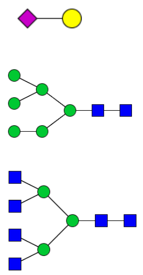	Saeland et al, 2012
CRC tissues/ MALDI-TOF/MS, HILIC CHROMATOGRAPHY	Comparison between CRC tumour and paired normal tissues	Increase of high-mannose, sulfated, paucimannosidic and sLewis X(sLe^{x}), decrease of bisecting compositions in tumour	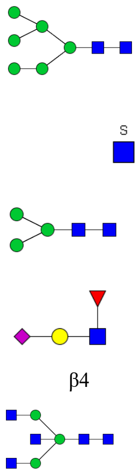	Balog et al, 2012
CRC tissues/Lectin blot	Comparison between CRC tumour and paired normal tissues	Increase of α2,3 sialylated residues	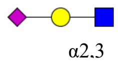	Fukasawa et al, 2013
CRC tissues and cell lines/LC-MS	Comparison between CRC tumour and cell lines (SW1116, SW480, SW620, SW837, LS174)	Increase of high-mannose in tumour and cell lines	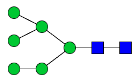	Chik et al, 2014
Cell lines/MS	N-glycans analysis of three cell lines (LIM 1215, LIM 1819 and LIM2405)	Increase of high-mannose and α2,6-sialylated N-glycans in all cell lines. Increase of bisecting compositions in LIM 1215 and increase of α2,3 sialylated compositions in LIM2405	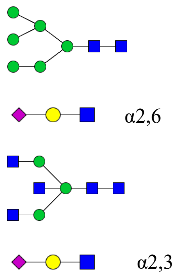	Sethi et al, 2014

CRC tissues/MALDI-TOF/MS	Comparison of N-glycans among tumour tissue, adenoma and paired normal tissue of rectal tumours	Increase of small mannose, paucimannosidic and sialylated compositions in CRC tissue in relation to adenoma. Worst prognosis in tumours with increased paucimannosidic	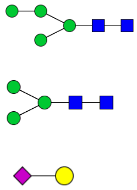	Kaprio et al, 2015
CRC tissues/	Comparison between N-glycans in EGFR + and EGFR -tumour tissues with paired normal tissues	Increase of high-mannose, paucimannosidic, hybrid compositions and higher α2,6-sialylation.<br><br>High bisecting and low α2,3 sialylation in EGFR +	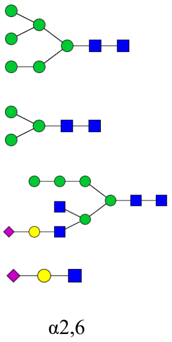<br>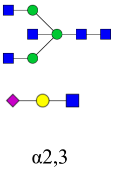	Sethi et al, 2015
CRC tissues/Lectin array	N-glycan profile in CEA of tumour tissues compared no paired normal tissues	Increase of fucose and mannose residues, decrease of branched and bisecting compositions in tumours. Decrease of mannose, galactose, N-Acetylglucosamine and N-Acetylgalactosamine in more advanced disease	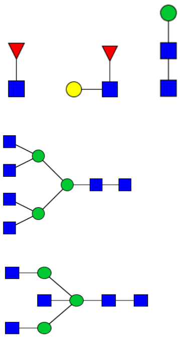	Zhao et al, 2018
CRC tissues/LC-MS	Comparison between N-glycosylation profile in tumour and paired normal tissues	Increase of high-mannose and bi-fucosylated and decrease of	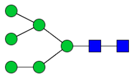	Zhang et al, 2019

		bisecting compositions in tumour. Decrease of bisecting compositions in more advanced disease	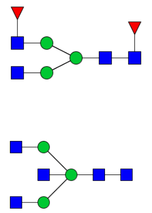	
CRC tissues/MALDI-TOF/MS	N-glycans profile in right- and left- sided tumour tissues and normal colon tissues of healthy individuals	Increase of acidic, paucimannosidic, high mannose N-glycans and decrease of bisecting compositions in tumour samples. No difference related to stage or sidedness	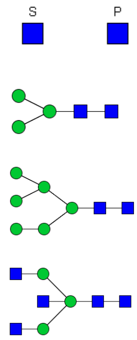	Holm et al, 2020
CRC tissue/MALDI imaging	Samples of stage II CRC tissue and peritumoral tissue	Increase of high mannose N-glycans and sialylation<br><br>Decrease of fucosylation and branched N-glycans	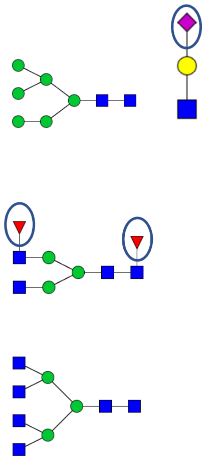	Boyaval et al,<br><br>2020
CRC tissue/MALDI-TOF/MS LC/MS	Comparison between N-glycosylation profile in tumour and paired normal tissues	Increase of high mannose, paucimannosidic, bi-antennary mono-galctosylated and branched N-glycans	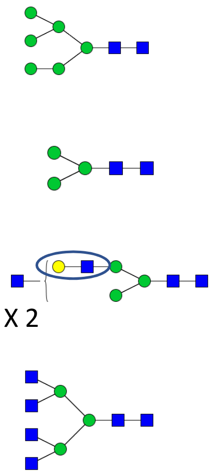	Present study,<br><br>2021
