# Supplementary material for: Identification of Differential N-Glycan Compositions in the Serum and Tissue of Colon Cancer Patients by Mass Spectrometry
Source: Biology (Basel). 2021 Apr 20;10(4):343. doi: 10.3390/biology10040343 (PMC8074232; doi:10.3390/biology10040343)
Supplement: Supplementary file 1 [file biology-10-00343-s001.zip › Supp_Material1_Tables S1_S2.pdf]

Identification of differential N-glycans in the serum and tissue of colon cancer patients by mass spectrometry

Marcelo de Melo Andrade Coura[1,2*], Eder Alves Barbosa[2,3], Guilherme Dotto Brand[,3], Carlos Bloch Jr[2], and Joao Batista de Sousa[1]

[1]Division of Colorectal Surgery, University Hospital of Brasilia, School of Medicine, University of Brasilia, Brasilia-DF, Brazil
[2]Laboratory of Mass Spectrometry, EMBRAPA Genetic Resources and Biotechnology, Brasilia-DF, Brazil
[3]Laboratory for the Synthesis and Analysis of Biomolecules, Institute of Chemistry, University of Brasilia, Brasilia-DF, Brazil

Supplemental material 1

Table S1. Clinicodemographic characteristics of healthy individuals (control group) (n=11)

Individuals	Genre	Age (years)	Indication	Colonoscopy
1	F	67	SCREENING	Normal
2	F	68	SCREENING	Diverticular disease
3	F	53	SCREENING	Normal
4	F	50	SCREENING	Normal
5	M	58	SCREENING	Diverticular disease
6	M	55	SCREENING	Normal
7	M	56	SCREENING	Normal
8	M	57	SCREENING	Normal
9	M	61	SCREENING	Diverticular disease
10	M	61	SCREENING	Normal
11	F	52	SCREENING	Normal

Table S2. N-glycan number, structure, accurate mass, mass used for quantification, corresponding adduct and the heavy reference number used for LC-MS and MALDI MS quantification methods.

Number	Proposed structure, theoretical mass and composition	Mass used for LCMS based quantification [a]	Adduct	Serum Heavy reference [b]		Structure confirmed by MS/MS data [c]
				LC-MS	MALDI	
**1**	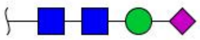 m/z: 1124.5572 [MONO,perMe,Na,0,freeEnd] HexNAc2Hex1NeuAc1	1124.5572	[M+Na]$^+$	13	-	-
**2**	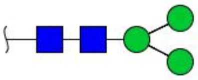 m/z: 1171.5831 [MONO,perMe,Na,0,freeEnd] HexNAc2Hex3	1171.5831	[M+Na]$^+$	13	-	-
**3**	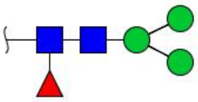 m/z: 1345.6723 [MONO,perMe,Na,0,freeEnd] HexNAc2Hex3Fuc1	1345.6723	[M+Na]$^+$	13	-	-
**4***	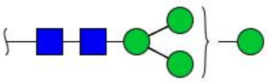 m/z: 1375.6828 [MONO,perMe,Na,0,freeEnd] HexNAc2Hex4	1375.6828*	[M+Na]$^+$	13	-	-
**5**	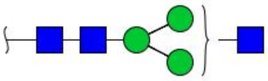 m/z: 1416.7094 [MONO,perMe,Na,0,freeEnd] HexNAc3Hex3	1416.7094	[M+Na]$^+$	13	-	✓✓

**6**	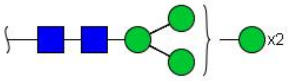 m/z: 1579.7826 [MONO,perMe,Na,0,freeEnd] HexNAc2Hex5	1579.7826	[M+Na]$^{+}$	52	6	✓
**7**	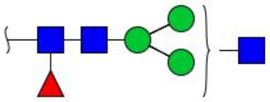 n/z: 1590.7986 [MONO,perMe,Na,0,freeEnd] HexNAc3Hex3Fuc1	1590.7986	[M+Na]$^{+}$	27	-	-
**8**	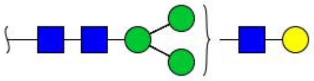 n/z: 1620.8091 [MONO,perMe,Na,0,freeEnd] HexNAc3Hex4	1620.8090	[M+Na]$^{+}$	19	-	*
**9**	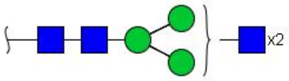 m/z: 1661.8357 [MONO,perMe,Na,0,freeEnd] HexNAc4Hex3	842.4125*	[M+2Na]$^{+}$	13	-	✓✓
**10**	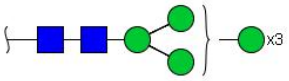 n/z: 1783.8824 [MONO,perMe,Na,0,freeEnd] HexNAc2Hex6	903.4358	[M+2Na]$^{2+}$	52	6	✓✓
**11**	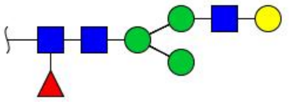 m/z: 1794.8984 [MONO,perMe,Na,0,freeEnd] HexNAc3Hex4Fuc1	908.9438	[M+2Na]$^{2+}$	27	-	-

**12**	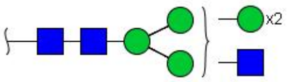<br>m/z: 1824.9089 [MONO,perMe,Na,0,freeEnd]<br>HexNAc3Hex5	1824.9089	$[M+Na]^{+}$	52	-	-
**13**	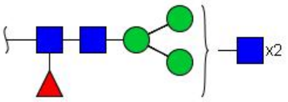<br>m/z: 1835.9249 [MONO,perMe,Na,0,freeEnd]<br>HexNAc4Hex3Fuc1	929.4571	$[M+2Na]^{2+}$	13	13	✓✓
**14**	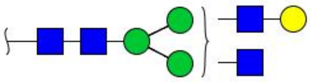<br>m/z: 1865.9355 [MONO,perMe,Na,0,freeEnd]<br>HexNAc4Hex4	1865.9355	$[M+Na]^{+}$	19	**19**	✓✓
**15**	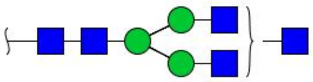<br>m/z: 1906.9620 [MONO,perMe,Na,0,freeEnd]<br>HexNAc5Hex3	964.9756	$[M+2Na]^{2+}$	13	-	-
**16**	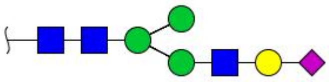<br>m/z: 1981.9828 [MONO,perMe,Na,0,freeEnd]<br>HexNAc3Hex4NeuAc1	1002.4860	$[M+2Na]^{2+}$	16	-	✓✓
**17**	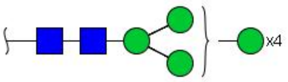<br>m/z: 1987.9821 [MONO,perMe,Na,0,freeEnd]<br>HexNAc2Hex7	1005.4857*	$[M+2Na]^{2+}$	52	6	✓✓

**18**	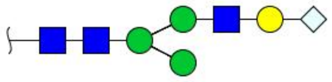 m/z: 2011.9934 [MONO,perMe,Na,0,freeEnd] HexNAc3Hex4NeuGc1	1017.4913	[M+2Na]$^{2+}$	35	-	-
**19**	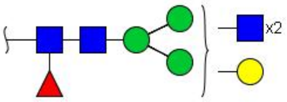 n/z: 2040.0247 [MONO,perMe,Na,0,freeEnd] HexNAc4Hex4Fuc1	1031.5070	[M+2Na]$^{2+}$	19	19	✓✓
**20**	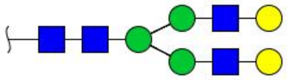 m/z: 2070.0352 [MONO,perMe,Na,0,freeEnd] HexNAc4Hex5	1046.5122	[M+2Na]$^{2+}$	52	27	✓✓
**21**	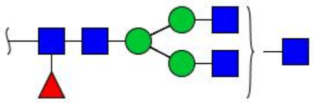 m/z: 2081.0512 [MONO,perMe,Na,0,freeEnd] HexNAc5Hex3Fuc1	1052.0202	[M+2Na]$^{2+}$	13	13	-
**22**	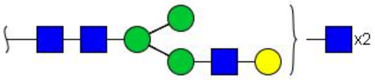 n/z: 2111.0618 [MONO,perMe,Na,0,freeEnd] HexNAc5Hex4	1067.0255	[M+2Na]$^{2+}$	19	-	*
**23**	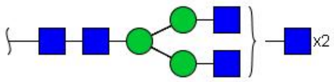 m/z: 2152.0883 [MONO,perMe,Na,0,freeEnd] HexNAc6Hex3	1087.5388*	[M+2Na]$^{2+}$	13	-	✓

**24**	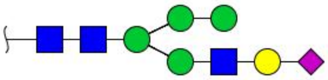 m/z: 2186.0826 [MONO,perMe,Na,0,freeEnd]<br>HexNAc3Hex5NeuAc1	1104.5359*	[M+2Na]$^{2+}$	52	35	✓✓
**25**	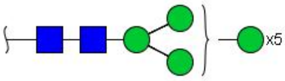 m/z: 2192.0819 [MONO,perMe,Na,0,freeEnd]<br>HexNAc2Hex8	1107.5356	[M+2Na]$^{2+}$	52	6	✓✓
**26**	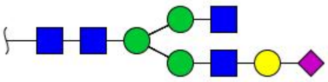 m/z: 2227.1091 [MONO,perMe,Na,0,freeEnd]<br>HexNAc4Hex4NeuAc1	1125.0492	[M+2Na]$^{2+}$	26	35	✓✓
**27**	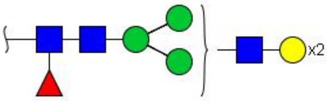 m/z: 2244.1245 [MONO,perMe,Na,0,freeEnd]<br>HexNAc4Hex5Fuc1	1133.5568	[M+2Na]$^{2+}$	27	27	✓✓
**28**	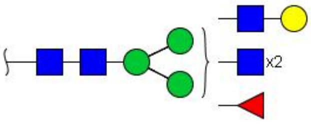 m/z: 2285.1510 [MONO,perMe,Na,0,freeEnd]<br>HexNAc5Hex4Fuc1	1154.0701	[M+2Na]$^{2+}$	19	19	✓
**29**	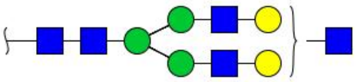 m/z: 2315.1616 [MONO,perMe,Na,0,freeEnd]<br>HexNAc5Hex5	1169.0754	[M+2Na]$^{2+}$	27	-	-

**30**	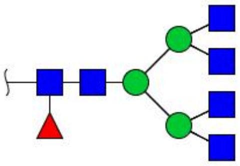<br>m/z: 2326.1776 [MONO,perMe,Na,0,freeEnd]<br>HexNAc6Hex3Fuc1	1174.5834	$[M+2Na]^{2+}$	13	-	-
**31**	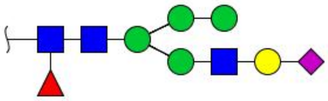<br>m/z: 2360.1718 [MONO,perMe,Na,0,freeEnd]<br>HexNAc3Hex5Fuc1NeuAc1	1191.5805	$[M+2Na]^{2+}$	27	-	✓✓
**32**	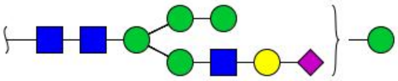<br>m/z: 2390.1824 [MONO,perMe,Na,0,freeEnd]<br>HexNAc3Hex6NeuAc1	1206.5858	$[M+2Na]^{2+}$	52	35	✓✓
**33**	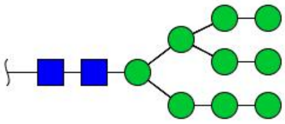<br>m/z: 2396.1817 [MONO,perMe,Na,0,freeEnd]<br>HexNAc2Hex9	1209.5855	$[M+2Na]^{2+}$	52	6	✓✓
**34**	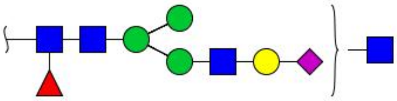<br>m/z: 2401.1983 [MONO,perMe,Na,0,freeEnd]<br>HexNAc4Hex74Fuc1NeuAc1	1220.0808	$[M+Na+K]^{2+}$	34	-	✓✓

**35**	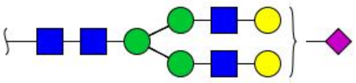<br>m/z: 2431.2089 [MONO,perMe,Na,0,freeEnd]<br>HexNAc4Hex5NeuAc1	1227.0991	$[M+2Na]^{2+}$	35	35	✓✓
**36**	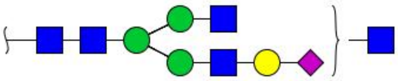<br>m/z: 2472.2355 [MONO,perMe,Na,0,freeEnd]<br>HexNAc5Hex4NeuAc1	1247.6123	$[M+2Na]^{2+}$	52	-	✓✓
**37**	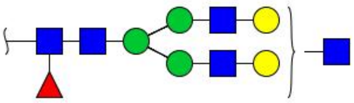<br>m/z: 2489.2508 [MONO,perMe,Na,0,freeEnd]<br>HexNAc5Hex5Fuc1	1256.1200	$[M+2Na]^{2+}$	27	27	-
**38**	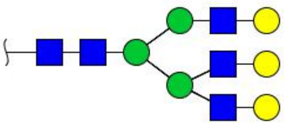<br>m/z: 2519.2613 [MONO,perMe,Na,0,freeEnd]<br>HexNAc5Hex6	1271.1253	$[M+2Na]^{2+}$	72	-	-
**39**	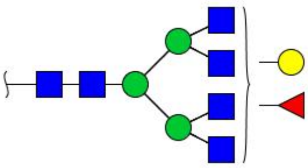<br>m/z: 2530.2773 [MONO,perMe,Na,0,freeEnd]<br>HexNAc6Hex4Fuc1	1276.6333	$[M+2Na]^{2+}$	27	-	-

**40**	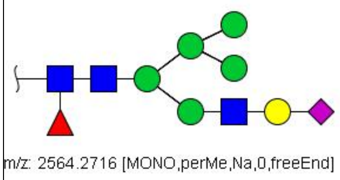 HexNAc3Hex6Fuc1NeuAc1	1293.6304*	[M+2Na]$^{2+}$	27	-	✓✓
**41**	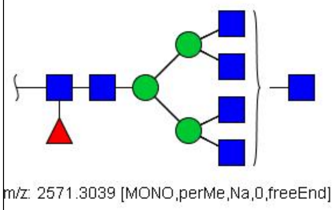 HexNAc7Hex3Fuc1	1297.1465	[M+2Na]$^{2+}$	75	-	*
**42**	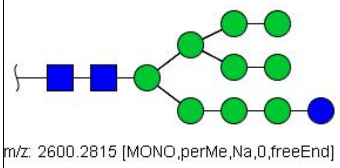 HexNAc2Hex10	1311.6353	[M+2Na]$^{2+}$	52	-	✓
**43**	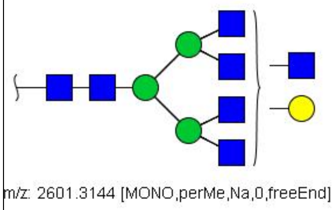 HexNAc7Hex4	1312.1518	[M+2Na]$^{2+}$	72	-	✓
**44**	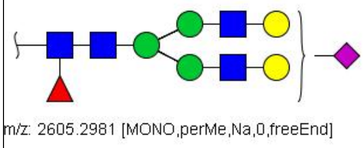 HexNAc4Hex5Fuc1NeuAc1	1314.1437	[M+2Na]$^{2+}$	44	58	✓✓

**45**	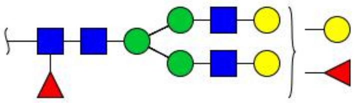<br>m/z: 2622.3134 [MONO,perMe,Na,0,freeEnd]<br>HexNAc4Hex6Fuc2	1322.6513	$[M+2Na]^{2+}$	27	-	-
**46**	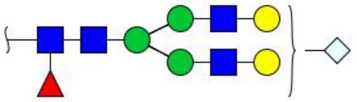<br>m/z: 2635.3087 [MONO,perMe,Na,0,freeEnd]<br>HexNAc4Hex5Fuc1NeuGc1	1329.149	$[M+2Na]^{2+}$	27	-	-
**47**	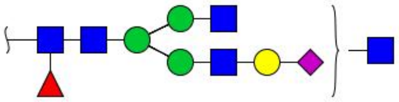<br>m/z: 2646.3247 [MONO,perMe,Na,0,freeEnd]<br>HexNAc5Hex4Fuc1NeuAc1	1334.6569*	$[M+2Na]^{2+}$	27	-	-
**48**	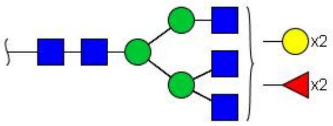<br>m/z: 2663.3400 [MONO,perMe,Na,0,freeEnd]<br>HexNAc5Hex5Fuc2	903.1061	$[M+3Na]^{3+}$	27	27	-
**49**	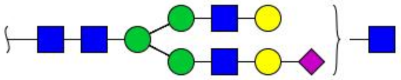<br>m/z: 2676.3352 [MONO,perMe,Na,0,freeEnd]<br>HexNAc5Hex5NeuAc1	907.4379*	$[M+3Na]^{3+}$	35	35	✓

**50**	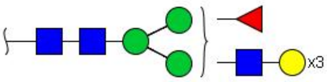 m/z: 2693.3506 [MONO,perMe,Na,0,freeEnd] HexNAc5Hex6Fuc1	913.1097	$[M+3Na]^{3+}$	27	-	✓
**51**	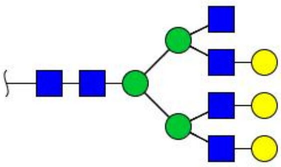 m/z: 2764.3877 [MONO,perMe,Na,0,freeEnd] HexNAc6Hex6	936.7887	$[M+3Na]^{3+}$	72	35	✓
**52**	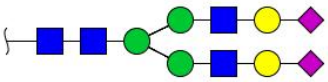 m/z: 2792.3826 [MONO,perMe,Na,0,freeEnd] HexNAc4Hex5NeuAc2	1407.6859	$[M+2Na]^{2+}$	52	52	✓✓
**53**	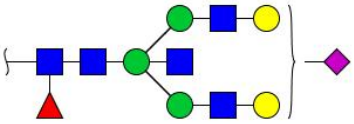 m/z: 2850.4244 [MONO,perMe,Na,0,freeEnd] HexNAc5Hex5Fuc1NeuAc1	965.4676	$[M+3Na]^{3+}$	27	35	✓✓
**54**	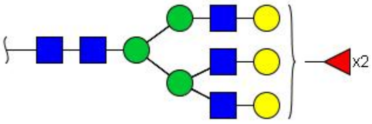 m/z: 2867.4398 [MONO,perMe,Na,0,freeEnd] HexNAc5Hex6Fuc2	971.1394	$[M+3Na]^{3+}$	27	-	-

**55**	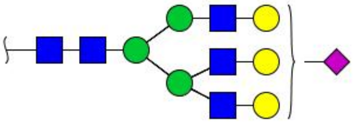<br>m/z: 2880.4350 [MONO,perMe,Na,0,freeEnd]<br>HexNAc5Hex6NeuAc1	975.4711	[M+3Na]$^{3+}$	72	-	✓✓
**56**	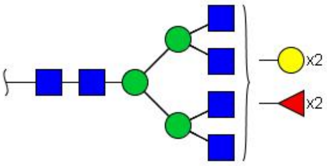<br>m/z: 2908.4663 [MONO,perMe,Na,0,freeEnd]<br>HexNAc6Hex5Fuc2	984.8149	[M+3Na]$^{3+}$	27	-	✓
**57**	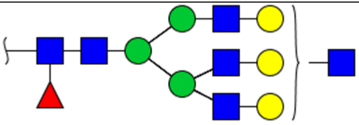<br>m/z: 2938.4769 [MONO,perMe,Na,0,freeEnd]<br>HexNAc6Hex6Fuc1	994.8184	[M+3Na]$^{3+}$	27	-	✓
**58**	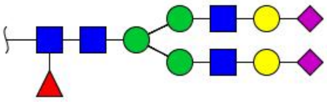<br>m/z: 2966.4718 [MONO,perMe,Na,0,freeEnd]<br>HexNAc4Hex5Fuc1NeuAc2	1004.1501	[M+3Na]$^{3+}$	27	58	✓✓
**59**	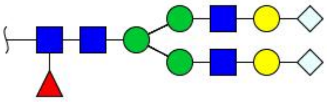<br>m/z: 3026.4929 [MONO,perMe,Na,0,freeEnd]<br>HexNAc4Hex5Fuc1NeuGc1	1532.728	[M+Na+K]$^{2+}$	27	-	-
**60**	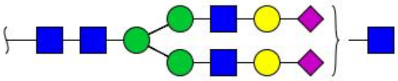<br>m/z: 3037.5089 [MONO,perMe,Na,0,freeEnd]	1027.8291	[M+3Na]$^{3+}$	52	-	-

	HexNAc5Hex5NeuAc2					
**61**	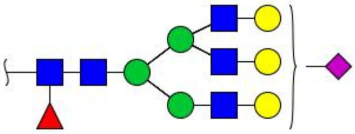<br>m/z: 3054.5242 [MONO,perMe,Na,0,freeEnd]<br>HexNAc5Hex6Fuc1NeuAc1	1033.5009*	[M+3Na]$^{3+}$	27	-	✓
**62**	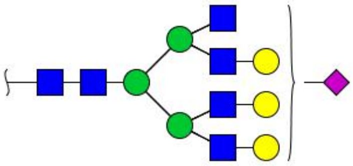<br>n/z: 3125.5613 [MONO,perMe,Na,0,freeEnd]<br>HexNAc6Hex6NeuAc1	1057.1799	[M+3Na]$^{3+}$	72	-	-
**63**	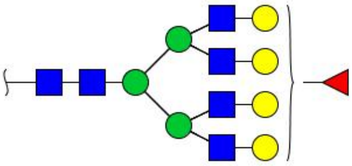<br>m/z: 3142.5766 [MONO,perMe,Na,0,freeEnd]<br>HexNAc6Hex7Fuc1	1062.8517	[M+3Na]$^{3+}$	27	-	-
**64**	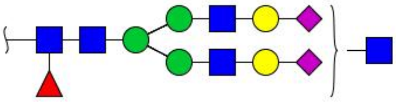<br>m/z: 3211.5981 [MONO,perMe,Na,0,freeEnd]<br>HexNAc5Hex5Fuc1NeuAc2	1617.2937	[M+2Na]$^{2+}$	27	64	✓
**65**	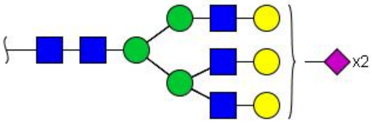<br>m/z: 3241.6087 [MONO,perMe,Na,0,freeEnd]	1095.8624	[M+3Na]$^{3+}$	72	52	-

	HexNAc5Hex6NeuAc2					
**66**	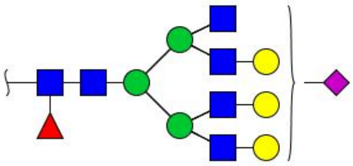<br>m/z: 3299.6505 [MONO,perMe,Na,0,freeEnd]<br>HexNAc6Hex6Fuc1NeuAc1	1115.2096	[M+3Na]$^{3+}$	27	-	✓
**67**	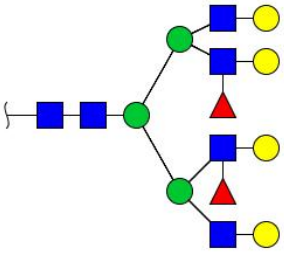<br>n/z: 3316.6659 [MONO,perMe,Na,0,freeEnd]<br>HexNAc6Hex7Fuc2	1669.8275*	[M+2Na]$^{2+}$	27	-	✓
**68**	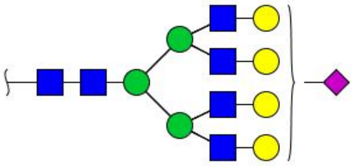<br>m/z: 3329.6611 [MONO,perMe,Na,0,freeEnd]<br>HexNAc6Hex7NeuAc1	1125.2132	[M+3Na]$^{3+}$	80	-	-
**69**	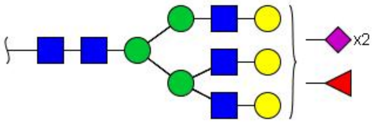<br><br>n/z: 3415.6979 [MONO,perMe,Na,0,freeEnd]	1153.8921	[M+3Na]$^{3+}$	27	52	✓

	HexNAc5Hex6Fuc1NeuAc2					
**70**	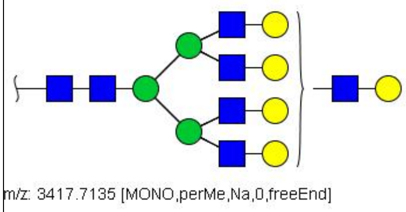 HexNAc7Hex8	1154.5640*	[M+3Na]$^{3+}$	80	-	✓
**71**	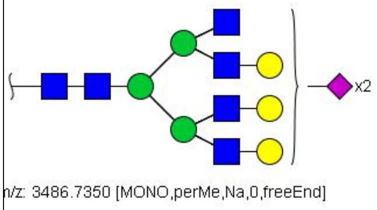 HexNAc6Hex6NeuAc2	1177.5711*	[M+3Na]$^{3+}$	72	-	-
**72**	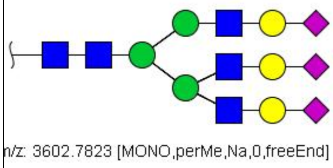 HexNAc5Hex6NeuAc3	1216.2536	[M+3Na]$^{3+}$	72	72	✓✓
**73**	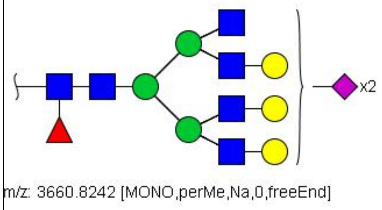 HexNAc6Hex6Fuc1NeuAc2	1235.6009*	[M+3Na]$^{3+}$	27	-	-

**74**	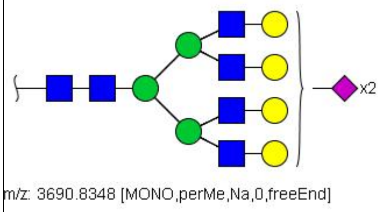<br>HexNAc6Hex7NeuAc2	1245.6044	$[M+3Na]^{3+}$	80	52	✓
**75**	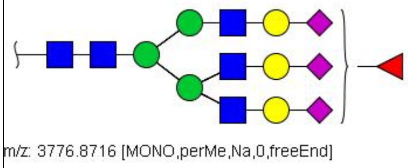<br>HexNAc5Hex6Fuc1NeuAc3	1274.2833	$[M+3Na]^{3+}$	27	75	✓✓
**76**	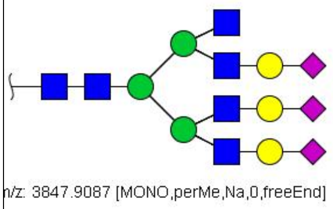<br>HexNAc6Hex6NeuAc3	1297.9624	$[M+3Na]^{3+}$	72	-	-
**77**	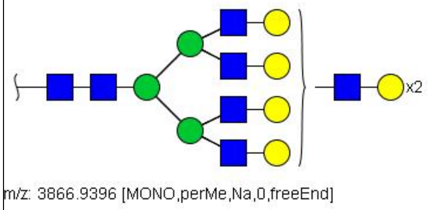<br>HexNAc8Hex9	1944.9644	$[M+2Na]^{2+}$	80	-	-

**78**	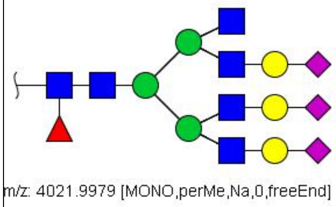<br>HexNAc6Hex6Fuc1NeuAc3	1355.9921	[M+3Na]$^{3+}$	27	-	-
**79**	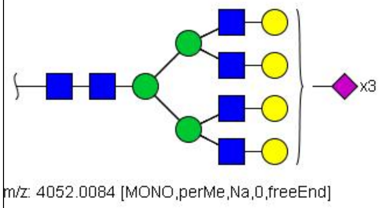<br>HexNAc6Hex7NeuAc3	1030.2440	[M+4Na]$^{4+}$	80	52	✓
**80**	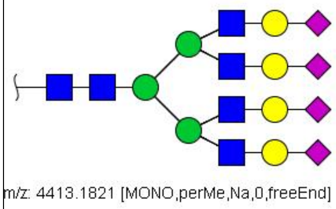<br>HexNAc6Hex7NeuAc4	1120.5374	[M+4Na]$^{4+}$	80	72	✓✓
**81**	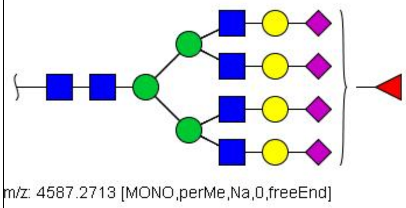<br>HexNAc6Hex7Fuc1NeuAc4	1164.0597	[M+4Na]$^{4+}$	80	75	✓

**82**	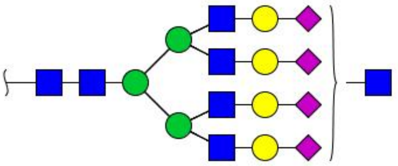 m/z: 4658.3084 [MONO,perMe,Na,0,freeEnd] HexNAc7Hex7NeuAc4	1181.8190*	[M+4Na]$^{4+}$	80	-	-
**83**	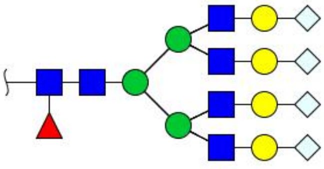 m/z: 4707.3136 [MONO,perMe,Na,0,freeEnd] HexNAc6Hex7Fuc1NeuGc4	1194.0703*	[M+4Na]$^{4+}$	80	-	-
**84**	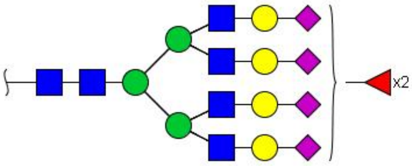 m/z: 4761.3605 [MONO,perMe,Na,0,freeEnd] HexNAc6Hex7Fuc2NeuAc4	1207.5820*	[M+4Na]$^{4+}$	80	-	-
**85**	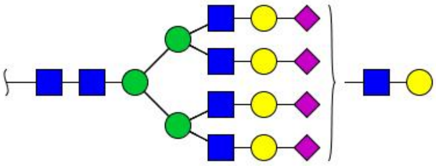 m/z: 4862.4082 [MONO,perMe,Na,0,freeEnd] HexNAc7Hex8NeuAc4	1232.8439*	[M+4Na]$^{4+}$	80	-	*

**86**	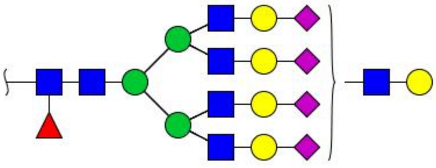 m/z: 5036.4974 [MONO,perMe,Na,0,freeEnd] HexNAc7Hex8Fuc1NeuAc4	1276.3663*	[M+4Na]$^{4+}$	80	-	✓
**87**	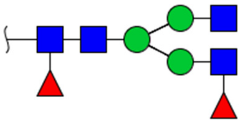 m/z: 2010.0141 [MONO,perMe,Na,0,freeEnd] HexNAc4Hex3Fuc2	1016.5017	[M+2Na]$^{2+}$	13	-	✓✓
**88**	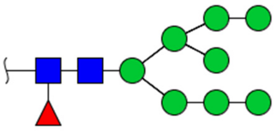 m/z: 2366.1711 [MONO,perMe,Na,0,freeEnd] HexNAc2Hex8Fuc1	1194.5802	[M+2Na]$^{2+}$	52	-	-
**89**	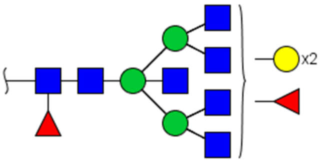 m/z: 3153.5926 [MONO,perMe,Na,0,freeEnd] HexNAc7Hex5Fuc2	1066.5237	[M+3Na]$^{3+}$	80	-	-
**90**	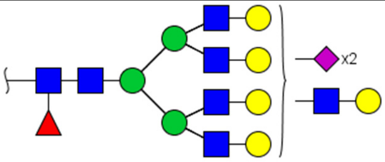 m/z: 4314.1501 [MONO,perMe,Na,0,freeEnd] HexNAc7Hex8Fuc1NeuAc2	1453.3762	[M+3Na]$^{3+}$	90	-	-

[a] MALDI MS based quantification was performed using masses corresponding to the [M+Na]$^{+}$ adducts only;

[b] Quantification of N-glycans isolated from tissues were performed using the heavy glycan of the same composition;

[c] one check indicates that permethylated structure was confirmed while double check indicates that both structures (permethylated and perdeuterated) were confirmed, while asterisk indicate that only perdeuterated structure was confirmed.

.

Legend: ■N-acetyl glucosamine (GlcNac), ● Mannose (Man), ● Galactose (Gal), ◀ Fucose (Fuc), ♦ Sialic acid (NeuAc).
